# A Report of Two Cases of Tuberculosis With Bronchial Anthracofibrosis: Diagnostic Challenges and Therapeutic Approaches

**DOI:** 10.7759/cureus.71517

**Published:** 2024-10-15

**Authors:** Berti Sahaya Louis, Abdul Majeed Arshad, Koushik Muthu Raja, Irfan Ismail Ayub, Thangaswamy Dhanasekar

**Affiliations:** 1 Pulmonology and Critical Care, Sri Ramachandra Institute of Higher Education and Research, Chennai, IND

**Keywords:** active pulmonary tuberculosis, bronchial anthracofibrosis, bronchoscopy, heterogeneous opacities, tuberculosis

## Abstract

A clinical entity that was reported less than ten years ago, bronchial anthracofibrosis is marked by multifocal bronchial lumen constriction and anthracotic coloring of the bronchial mucosa. There is various etiology associated with “black bronchoscopy”. A 47-year-old woman with no known co-morbidities reported having a three-month history of coughing up sputum. She had been complaining of intermittent fever and weight loss for a month. Right upper zone heterogeneous opacity was visible on the chest X-ray. Additionally, she reported unilateral leg pain and swelling, which a Doppler examination revealed to be right deep vein thrombosis (DVT). PET-CT scan results for the patient revealed persistently active bilateral lung Koch's infection accompanied by healing mediastinal lymphadenopathy. Analyses of sputum were not helpful. Another patient, a 57-year-old male patient with a month-long period of severe weight loss, loss of appetite, and sporadic cough with expectoration was reported. He was not a smoker and had no known comorbidities. An unremarkable general examination was followed by systemic examinations that revealed crepitation in the interscapular and right mammary regions. The right lower zone of a chest X-ray revealed heterogeneous opacities. Both patients underwent bronchoscopy, which showed the presence of bronchial anthracosis and anthracofibrosis. Although the most common cause of these black pigmentations is dust exposure, tuberculosis can also present as bronchial anthracofibrosis. Enhanced diagnostic strategies, including advanced imaging techniques and bronchoscopic evaluation, are essential to distinguish between these conditions accurately and to guide treatment.

## Introduction

A diagnosis of bronchial anthracofibrosis (BAF) is made through bronchoscopy, which is characterized by the constriction or obliteration of the proximal airway, as well as bluish-black mucosal anthracotic pigmentation. This condition may manifest with or without prior exposure to occupational dust; however, it is more frequently observed in individuals who have inhaled biomass fuel fumes for an extended period. Because of the deposits of carbon, silica, and other inhaled contaminants, anthracosis is marked by a black discoloration of the tracheobronchial tree. Chronic BAF refers to any kind of anthracosis that results in the complete destruction or obstruction of the lungs. The terminology was first used by Chung et al. in 1998 [1,2]. Although this illness may manifest regardless of a history of occupational dust exposure, it primarily affects individuals who have a documented record of protracted exposure to biomass fuel pollution [3]. However, this common bronchoscopic finding can also be found in people with pulmonary tuberculosis, especially those who are living in South Korea, Iran, and India. Most often impacted is the right middle lobe, which is commonly linked to tuberculosis [4]. Bronchoscopic visualization of the above-mentioned narrowing and pigmentation confirms the diagnosis. Other clinical diseases like pneumonia, cancer, and chronic obstructive pulmonary disease have all been linked to BAF. Previously believed to be the cause of BAF, tuberculosis is now recognized as a contributing factor [5]. Given that BAF is frequently associated with tuberculosis, patients with endobronchial tuberculosis (EBTB) and BAF appear to be experiencing distinct pathological processes that impact the same airway mucosa.

## Case presentation

Case one

**Figure 1:**
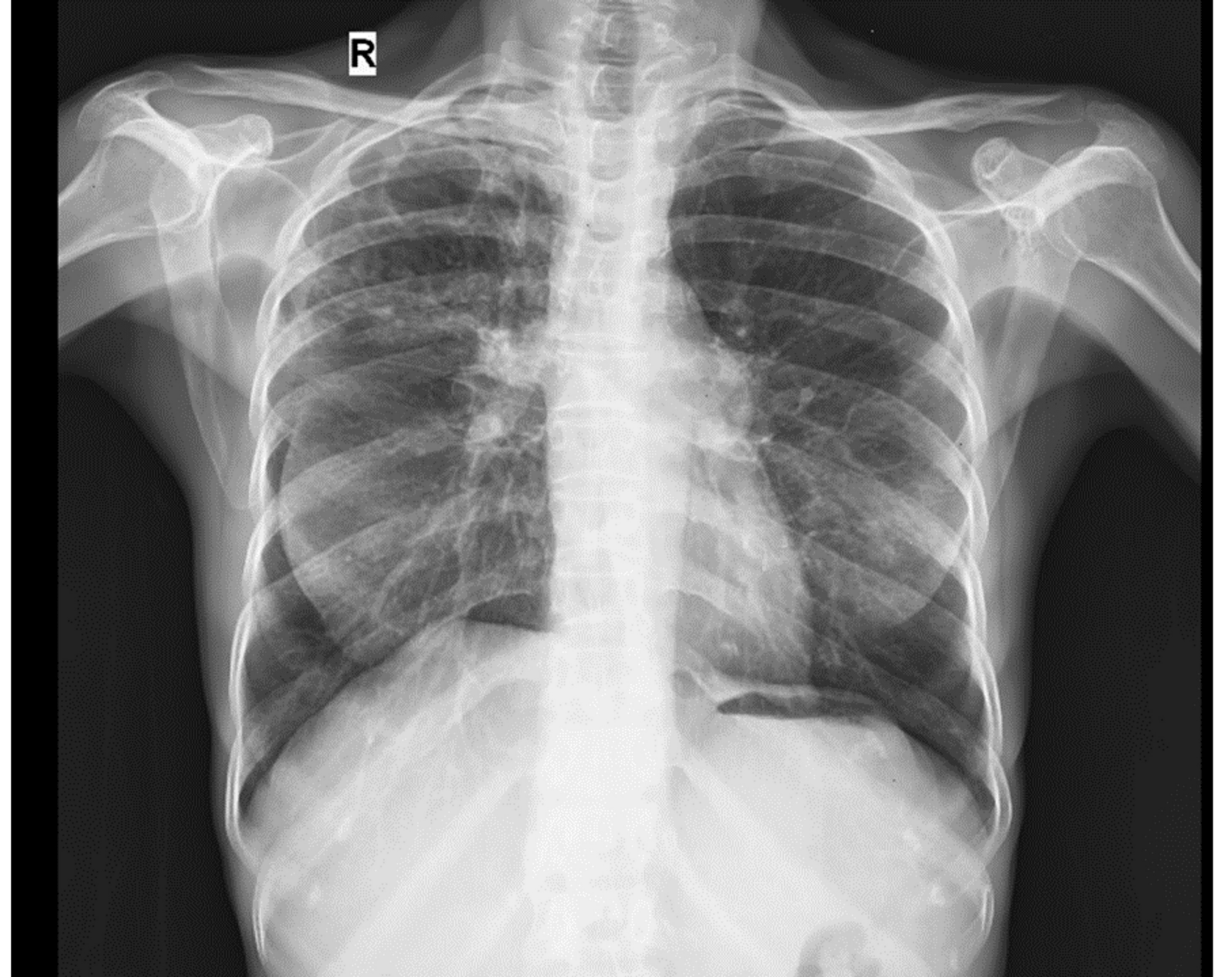
Chest X-ray showing bilateral heterogeneous opacities in the lungs

A 47-year-old female with no known co-morbidity and no significant exposure to smoking or biomass exposure presented with a history of cough with sputum for three months. She complained of on-and-off fever and loss of weight for one month's duration. Her blood investigations were unremarkable. A chest X-ray showed the right upper zone heterogenous opacity as shown in Figure 1. She also complained of unilateral leg swelling and pain, which revealed right deep vein thrombosis (DVT) in the Doppler study. The patient underwent PET-CT, which showed chronically active Koch’s infection in bilateral lungs with healing mediastinal lymphadenopathy. Sputum analyses were noncontributory.

**Figure 2:**
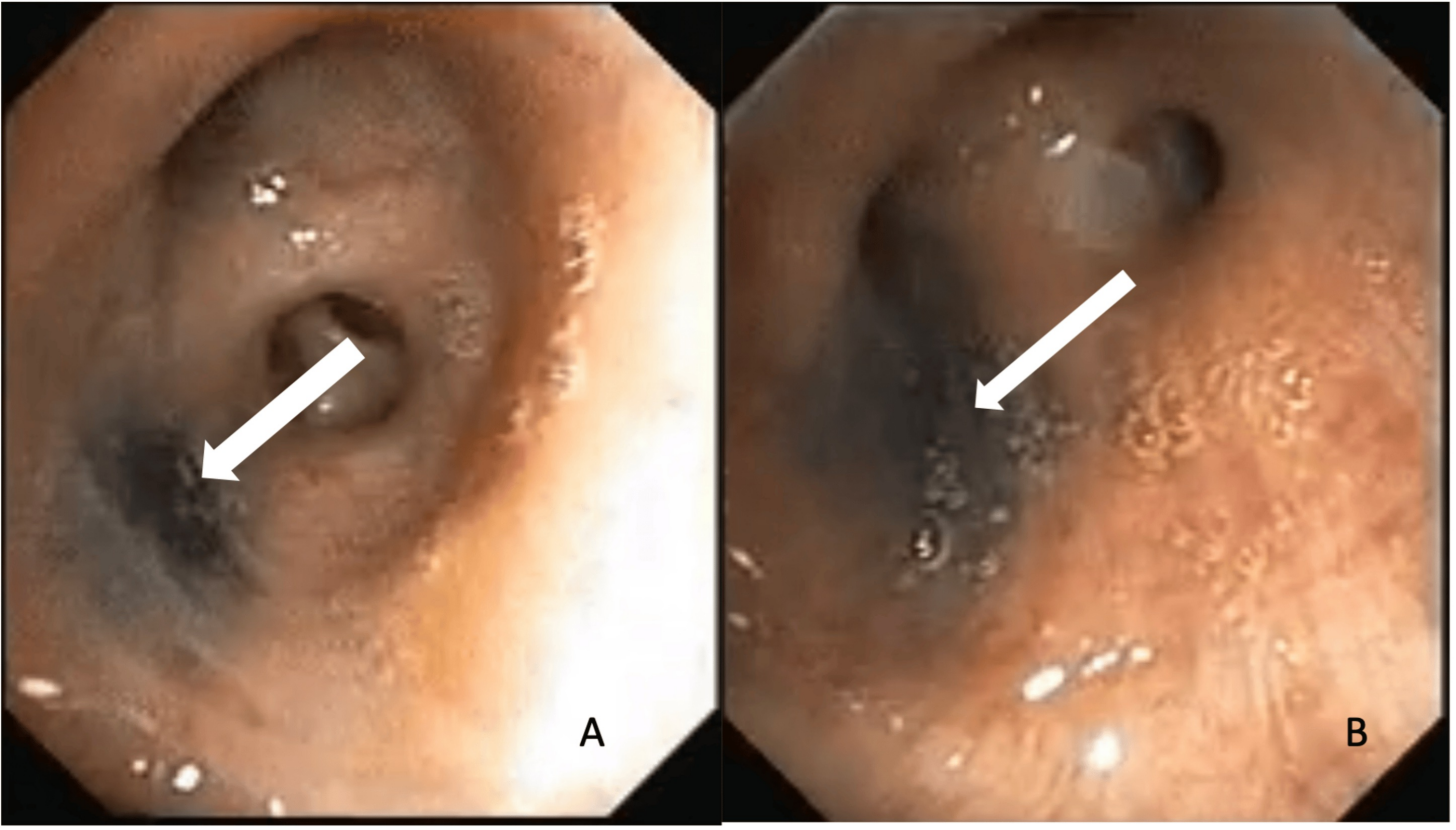
Bronchoscopy showing hyperpigmented patch in the right middle lobe bronchus

Subsequently, she underwent flexible bronchoscopy, which showed non-inflamed bronchial mucosa with many hyperpigmented patches with indistinct margins throughout the tracheobronchial tree (Figure 2). There was no architectural distortion, and the rest of the tracheobronchial tree was normal. A bronchoalveolar lavage cartridge-based nucleic acid amplification test (CBNAAT) was done. Mycobacterium tuberculosis (MTB) was detected, rifampicin (RIF) resistance was not detected, and malignant cells, fungal smear, and fungal culture were negative. The patient was started on rifampicin, isoniazid, pyrazinamide, and ethambutol (RIPE) therapy. Six months of antituberculosis therapy is planned for her, and she is on regular follow-up.

Case two

**Figure 3:**
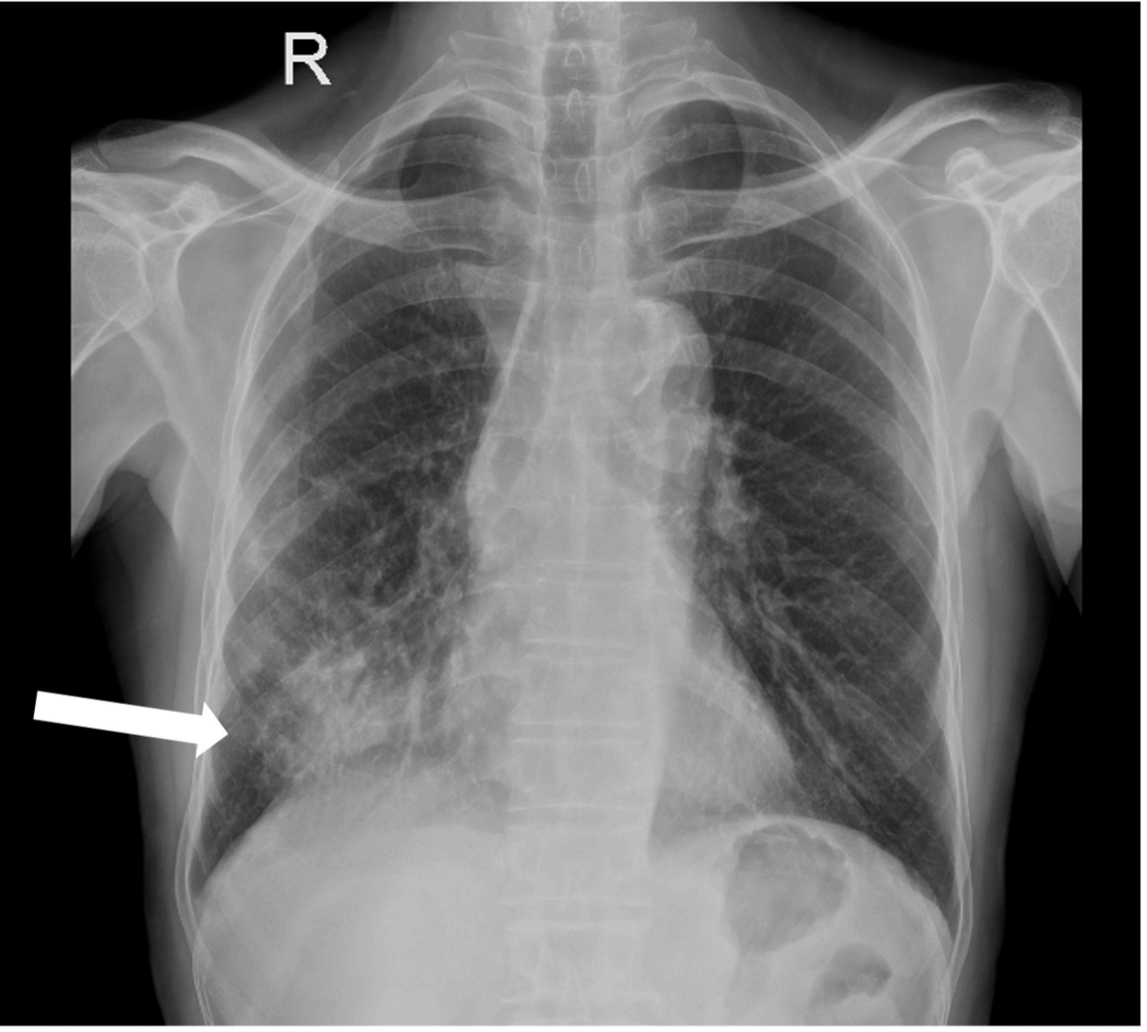
X-ray showing right lower zone heterogeneous opacities

The second patient was a non-smoker 57-year-old male patient with no known comorbidities and no significant exposure history. He presented with significant weight loss, loss of appetite, and occasional cough with expectoration over the course of a month. A general examination was unremarkable and systemic examinations showed crepitation heard in the right mammary and interscapular area. The initial blood investigation was unremarkable. A chest X-ray showed heterogeneous opacities in the right lower zone as shown in Figure 3.

Differential diagnosis

We considered the following in the differential diagnoses: community-acquired pneumonia, aspiration pneumonia, post-obstructive pneumonia, pulmonary tuberculosis, fungal pneumonia, organizing pneumonia, and malignancy.

Bronchoscopy findings

**Figure 4:**
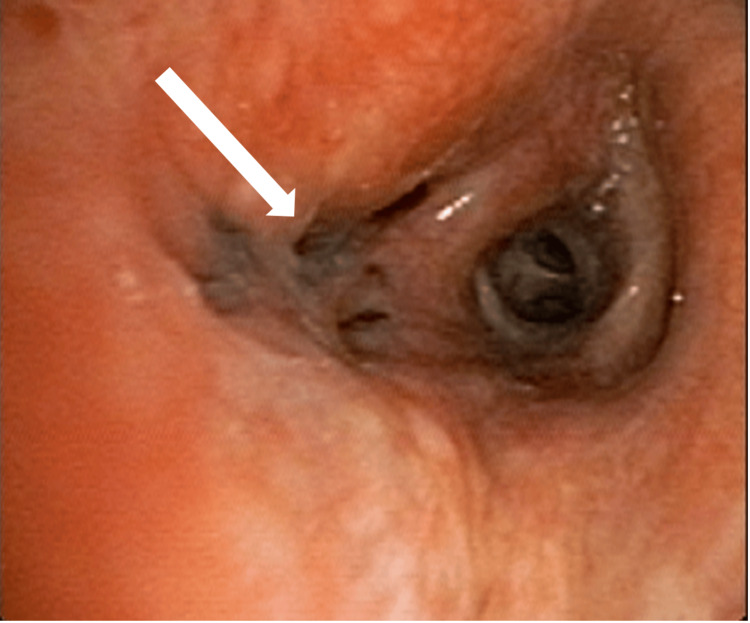
Bronchoscopy showing narrowing of the right middle lobe bronchus

A bronchoscopy was performed, and it revealed an endobronchial hyperpigmented lesion in the right middle lobe bronchus. The narrowing of the right middle lobe bronchus is shown in Figure 4. Multiple biopsies were taken, and a post-biopsy bronchial wash was sent for comprehensive analysis including CBNAAT MTB, fungal smear and culture, bacterial culture, and cytology.

**Figure 5:**
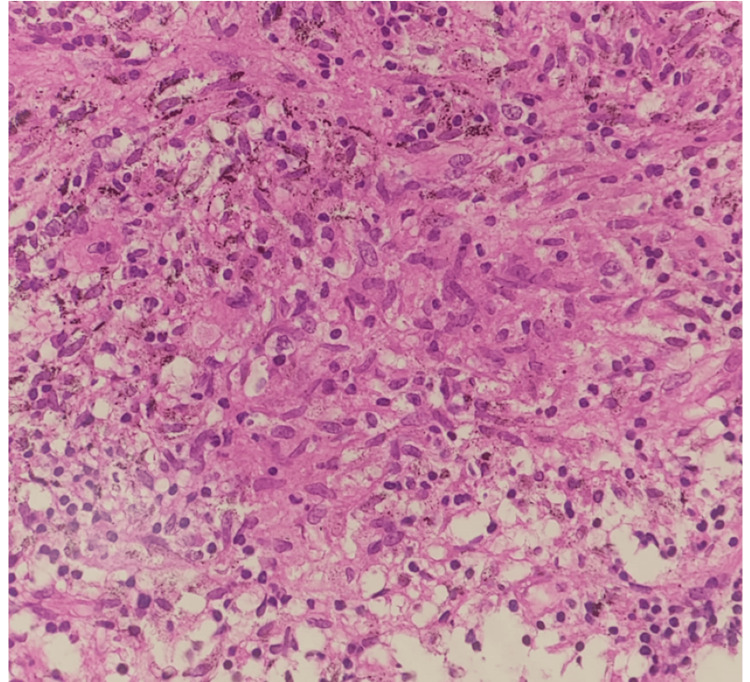
Histopathological examination (HPE) showing ill-defined epithelioid granuloma on 400x magnification

The bronchial wash analysis was carried out and CBNAAT MTB detected Mycobacterium tuberculosis. RIF resistance was not detected. Other test results were inconclusive. The endobronchial biopsy was sent for histopathological examination (HPE) and showed necrotizing granulomatous lesions as shown in Figure 5.

The patient was diagnosed with pulmonary tuberculosis and started on an antituberculosis drug. She was advised to undergo regular follow-up.

## Discussion

A retrospective analysis was conducted by Dang et al. [6] on the clinical aspects of two cases of BAF associated with tuberculosis. Both patients displayed signs of expectoration, along with a chronic cough. The bronchoscopy procedure revealed pigmentation in the bronchus mucosa and the occurrence of lumen stenosis. Verification of tuberculosis infection was achieved through the use of biopsies. Both of our case reports have documented similar findings. The treatment for tuberculosis led to significant symptom relief and no subsequent occurrence. It is plausible that BAF and tuberculosis can occur simultaneously. Establishing a presumptive diagnosis of tuberculosis infection is of utmost importance to avoid misdiagnosis in individuals with BAF.

A study conducted by Paulin et al. examined the case of a 76-year-old patient who presented symptoms of coughing, exhaustion, and a loud inspiratory wheeze persisting for several months. The results of the chest CT scan showed the existence of many sub-centimeter pulmonary nodules, bilateral lower lobe ground glass infiltrates, constriction of the proximal right main stem bronchus following bronchoscopy, stenosis of the right main stem bronchus, and flat, friable, darkly pigmented submucosal lesions. Smear microscopy revealed acid-fast bacilli in the bronchoalveolar lavage fluid of the right middle lobe, and culture confirmed the growth of MTB. After receiving anti-MTB treatment, the patient's symptoms subsided [7]. Similar observations were made in our second case, including the narrowing of the right middle lobe.

Jung et al. concluded from their study that although cavitation was infrequent, there was a high prevalence of lower lobe predominance, endobronchial involvement, lymphadenopathy, and internal low-density foci or focal contour bulging within atelectasis in active pulmonary tuberculosis with BAF. The CT findings of pulmonary TB with BAF differ significantly when contrasted with the conventional signs of pulmonary TB without BAF. Thus, it will be very beneficial for the early detection of TB to be aware of these characteristic CT findings of pulmonary TB in the presence of BAF [3]. CT findings given in our study showed healing mediastinal lymphadenopathy.

In a study conducted by Saman et al., it was found that the thorough assessment of individuals with respiratory problems still heavily relies on gathering a comprehensive exposure history [8]. The coexistence of BAF and pulmonary tuberculosis has been evidenced by this particular occurrence, which is further supported by explanations based on evidence. Future investigations should prioritize studying the long-term consequences of extended wood smoke exposure on the quality of life among patients with BAF, as assessed through routine clinical evaluation and spirometry monitoring.

A study conducted by Kunal and Shah highlights the tendency to overlook the need for an intrusive diagnostic technique for BAF when diagnosing tuberculosis in patients with a history of prolonged exposure to biomass fuel smoke. Instead, the focus is primarily on diagnosing pulmonary tuberculosis [9]. It is crucial to emphasize the diagnosis of BAF in these patients and eliminate the possibility of pulmonary tuberculosis. In one unit, there were four cases where individuals diagnosed with BAF also had a concurrent diagnosis of tuberculosis out of thirty-one cases. A cough was the most commonly reported symptom among the four patients. Among the individuals, 75% displayed consolidation on imaging, while one exhibited nodular lesions and another showed multifocal narrowing on high-resolution CT (HRCT), which indicates BAF. Positive results were obtained for Mycobacterium tuberculosis stain and bronchial aspirate culture in all four patients, and the GeneXpert rapid molecular diagnostic system (Cepheid, Sunnyvale, California, United States) yielded positive results in all three patients. There was no evidence of resistance to RIF.

A study was undertaken by Yoon et al. to clarify the findings of lung parenchyma-associated anthracofibrosis on CT [10]. A retrospective assessment was performed on the CT findings of 34 individuals who exhibited anthracofibrosis in the lung parenchyma. Isolated fibrotic lesions were identified in conjunction with the deposition of anthracotic pigmentation based on histologic analysis. The manifestation of anthracofibrosis in lung parenchyma is characterized by the presence of a nodule, mass, or fibrotic consolidation encompassed by lengthy spicules. BAF can manifest in different ways, affecting the small airways of the lung parenchyma. These findings show a strong correlation with our case.

Less than 10 years ago, Kala et al. conducted a study that identified a clinical entity called BAF, which was characterized by a case of tuberculosis and presented as a middle lobe syndrome [11]. This condition is distinguished by a multifocal constriction of the bronchial lumen and a bronchial mucosa that exhibits an anthracotic coloration. Tuberculosis is commonly associated with the right middle lobe, which is primarily affected. The condition usually targets elderly women who have never smoked but have a long-standing history of wood smoke exposure. We had a visit from a 65-year-old female patient who complained of a dry cough that had persisted for a month. The middle lobe syndrome, which was initially detected on the chest radiograph, was subsequently confirmed through a CT scan. Moreover, there was evidence of a constriction in the bronchus of the right middle lobe. This led to the emergence of suspicions regarding the presence of cancer. Fiberoptic bronchoscopy revealed the presence of anthracotic coloration, while acid-fast bacilli were observed in the bronchial aspirate. The culture of the aspirate revealed the growth of Mycobacterium tuberculosis. The patient’s recovery was attributed to the administration of antituberculosis treatment.

A systematic review carried out by Mirsadraee et al. aimed to investigate the frequency of tuberculosis in anthracosis patients and non-anthracotic controls. Our meta-analysis included twelve papers with a combined sample size of 6280 individuals. Of these papers, eight were dedicated to studying anthracofibrosis, while the remaining four covered anthracosis in general. The occurrence of tuberculosis among all patients with anthracosis was found to be 22.5% (32.3% for anthracofibrosis and 16.6% for anthracosis), which significantly exceeded the prevalence observed in the control group. The cumulative odds ratio for tuberculosis in all anthracosis studies was found to be 3.16, showing a significantly higher risk when compared to the control group. The acquisition of this information was made possible through implementing a risk assessment procedure. The subgroup analysis revealed a significantly higher cumulative odds ratio of tuberculosis (3.28) in the anthracofibrosis subgroups compared with the general group of anthracosis (2.85) [12].

The performance of sputum smears and culture tests for Mycobacterium tuberculosis is strongly recommended for all patients suspected of having endobronchial tuberculosis. Even in an ideal laboratory environment with thorough sputum analysis, the sputum smear results for acid-fast bacilli do not provide adequate information regarding parenchymal involvement. Recent investigations have shown that the range of sputum positivity in EBTB is between 16% and 53.3% [13].

A bronchoscopy biopsy ought to be carried out in every instance of anthracofibrosis. In addition to being cultured, the biopsy samples need to be assessed for acid-fast bacilli. Our study illustrated the potential relationship between endobronchial tuberculosis and anthracofibrosis.

## Conclusions

The coexistence of tuberculosis and BAF can obscure the clinical picture, making accurate diagnosis challenging. The distinctive pigmentation of BAF may be misinterpreted because of chronic dust exposure, necessitating careful differential diagnosis. Effective management of patients with both tuberculosis and BAF requires a multidisciplinary approach. Addressing both conditions simultaneously is crucial for optimizing patient outcomes and reducing the risk of complications. Further research is needed to explain the mechanisms linking tuberculosis and BAF, including potential environmental and genetic factors. Additionally, exploring targeted therapies for managing coexisting tuberculosis and BAF could improve treatment strategies.
